# Supplementary material for: REV-ERBα integrates colon clock with experimental colitis through regulation of NF-κB/NLRP3 axis
Source: Nat Commun. 2018 Oct 12;9:4246. doi: 10.1038/s41467-018-06568-5 (PMC6185905; doi:10.1038/s41467-018-06568-5)
Supplement: Supplementary file 1 — Supplementary Information [file 41467_2018_6568_MOESM1_ESM.pdf]

Supplementary Information

REV-ERBα Integrates Colon Clock with Experimental Colitis through Regulation of NF-κB/NLRP3 Axis

Wang et al.

Supplementary Table 1. Mouse primer sequences for quantitative real-time PCR (qPCR)

**Gene**	**Forward (5'-3' sequence)**	**Reverse (3'-5' sequence)**
Bmal1	CTCCAGGAGGCAAGAAGATTC	ATAGTCCAGTGGAAGGAATG
Bmal1 (knockout region)	AGCGACTTCATGTCTCCG	GCTCTTACTAGTCAGTGGCA
Dbp	ACATCTAGGGACACACCCAGTC	AAGTCTCATGGCCTGGAATG
Rev-erbα	TTTTTCGCCGGAGCATCCAA	ATCTCGGCAAGCATCCGTTG
Rev-erbα (knockout region)	TCAGCTACAACTCCACACCG	CCCTGGCGTAGACCATTCAG
Rev-erbβ	GGAGTTCATGCTTGTGAAGGCTGT	CAGACACTTCTTAAAGCGGCACTG
Clock	TCTGGATTCGCTGGCTAATGG	GACCTCCGCTGTGTCATCTT
Per2	CCACACTTGCCTCCGAAATA	ACTGCCTCTGGACTGGAAGA
Cry1	CCCAGGCTTTTCAAGGAATGGAAC	GCAGGGAGTTTGCATTCATTCGAG
Npas2	CAGGACTGGAAGCCATCATT	CTGATGTTGGAGGCATTAGATGGC
Rorα	GAGACCCCGCTGACCCA	TGACTGAGATACCTCGGCTG
IL-1α	CCGGGTGACAGTATCAGCAA	CTGGGTTGGATGGTCTCTTCC
IL-1β	AATGCCACCTTTTGACAGTGATG	AGCTTCTCCACAGCCACAAT
IL-18	TCAAAGTGCCAGTGAACCCC	GGTCACAGCCAGTCCTCTTAC
IL-6	ATCCAGTTGCCTTCTTGGGACTGA	TAAGCCTCCGACTTGTGAAGTGGT
Tnfα	AGGGTCTGGGCCATAGAACT	CCACCACGCTCTTCTGTCTAC
IFN-γ	GCTACACACTGCATCTTGGC	CATGTCACCATCCTTTTGCCAG
Nlrp3	ATTACCCGCCCGAGAAAGG	TCGCAGCAAAGATCCACACAG
ASC	CTGGAGTCGTATGGCTTGGAG	CAAAGTGTCCTGTTCTGGCTGTA
Casp1	ACAAGGCACGGGACCTATG	TCCCAGTCAGTCCTGGAAATG
p65	ACTGCCGGGATGGCTACTAT	TCTGGATTCGCTGGCTAATGG
p50	GGGGCCTGCAAAGGTTATC	TGCTGTTACGGTGCATACCC
18s	CGGACAGGATTGACAGATTGATAGC	TGCCAGAGTCTCGTTCGTTATCG

Supplementary Table 2. Oligonucleotide sequences for EMSA assays

Oligonucleotide	Forward (5'-3' sequence)	Reverse (3'-5' sequence)
Bmal1-RevRE	GATTGGTCGGAAAGTAGGTTAGTGGTGCGAC	GTCGCACCACTAACCTACTTTCCGACCAATC
Nlrp3- RevRE	GTGTCACAGTGACCCCTATATATTTATCT	AGATAAATATATAGGGGTCACTGTGACAC
Nlrp3- RevRE -mu	GTGTCACAGGCTAGGCCCCCCCTTTATCT	AGATAAAGGGGGGGCCTAGCCTGTGACAC
P65- RevRE	TTCACGGTGTGACCCTAGCTGGTTCCGG	CCGGAACCAGCTAGGGTCACACCGTGAA
P65- RevRE -mu	TTCACGGTGTCAAAATACATGGTTCCGG	CCGGAACCATGTATTTTGACACCGTGAA

Supplementary Table 3. Primer sequences for ChIP assays

Gene	Forward (5'-3' sequence)	Reverse (3'-5' sequence)
Bmal1_ RevRE	GGAAAGTAGGTTAGTGGTGCGAC	AAGTCCGGCGCGGGTAAACAGG
Nlrp3_ RevRE	GCCAATCCGTCTTTGACAGTG	GCTCCAGTCCGTGTTCTCC
Nlrp3_Negative region	AATGCTTTTTGCGTTTTGCAGT	GACTCAGGAAGACAGGAGCC
P65_ RevRE	TTTCCTCATTGGAGCCTGGA	CACACATAGGTGCTGTCTGCT
P65_ Negative region	AATCTGGATGGGAAGAGCCT	GAAGGGGATGCAGGGGAC

Supplementary Table 4. Panther Pathway analysis for DEGs. The categories were listed based on the *p* values (*p*<0.05).

Term	Pathway ID	Count	Background number	*p* value
Angiogenesis	P00005	81	145	1.76E-05
Gonadotropin-releasing hormone receptor pathway	P06664	106	212	3.79E-05
Wnt signaling pathway	P00057	116	246	1.12E-04
Interleukin signaling pathway	P00036	50	83	1.77E-04
Inflammation mediated by chemokine and cytokine signaling pathway	P00031	102	214	2.08E-04
PDGF signaling pathway	P00047	61	113	3.55E-04
Integrin signalling pathway	P00034	74	147	4.44E-04
Cadherin signaling pathway	P00012	60	117	1.04E-03
Alzheimer disease-presenilin pathway	P00004	57	111	1.33E-03
Endothelin signaling pathway	P00019	42	74	1.34E-03
Huntington disease	P00029	52	111	8.48E-03
Muscarinic acetylcholine receptor 1 and 3 signaling pathway	P00042	29	53	9.94E-03
Oxytocin receptor mediated signaling pathway	P04391	29	55	1.42E-02
EGF receptor signaling pathway	P00018	47	102	1.47E-02
PI3 kinase pathway	P00048	24	43	1.52E-02
VEGF signaling pathway	P00056	31	61	1.66E-02

Term	Pathway ID	Count	Background number	*p* value
Notch signaling pathway	P00045	20	34	1.73E-02
FGF signaling pathway	P00021	45	99	1.97E-02
Histamine H1 receptor mediated signaling pathway	P04385	23	42	2.01E-02
p53 pathway	P00059	34	70	2.01E-02
5-Hydroxytryptamine degredation	P04372	14	21	2.16E-02
5HT2 type receptor mediated signaling pathway	P04374	31	63	2.27E-02
Insulin/IGF pathway-protein kinase B signaling cascade	P00033	19	33	2.33E-02
Alpha adrenergic receptor signaling pathway	P00002	12	17	2.45E-02
Blood coagulation	P00011	20	36	2.61E-02
Hedgehog signaling pathway	P00025	10	13	2.70E-02
Alzheimer disease-amyloid secretase pathway	P00003	29	59	2.71E-02
Circadian clock system	P00015	8	9	2.81E-02
Metabotropic glutamate receptor group II pathway	P00040	21	39	2.88E-02
Synaptic vesicle trafficking	P05734	15	25	3.26E-02
Apoptosis signaling pathway	P00006	45	104	3.49E-02
Plasminogen activating cascade	P00050	9	12	3.88E-02
Cytoskeletal regulation by Rho GTPase	P00016	30	65	4.35E-02
Muscarinic acetylcholine receptor 2 and 4 signaling pathway	P00043	24	50	4.96E-02
Thyrotropin-releasing hormone receptor signaling pathway	P04394	27	58	4.99E-02

Supplementary Table 5. Cosinor analyses of rhythmic expressions of clock genes in the colons of DSS-treated and normal mice. *p<0.05; n.s., not significant.

Group	Gene	Period(h)	Acrophase (°)	Robustness (%)	Mesor	Amplitude	
Normal	*Rev-erbα*	22.1	-196	89.3	0.7088	0.4833	*
	Npas2	24.8	-7	99.7	1.4988	0.9062	*
	Cry1	20.9	-9	92.0	2.2653	1.4229	*
	Per2	21,6	-334	89.9	4.1565	3.2354	*
	Clock	20.0	-17	76.3	1.8286	1.0584	*
	Dbp	22.8	-258	96.3	1.8592	1.7387	*
	Rev-erbβ	22.7	-259	97.5	1.6082	1.1407	*
	Bmal1	23.4	-28	96.6	1.3805	0.9900	*
	Rorα	20.0	-347	89.5	2.2811	1.0385	*
Colitis	*Rev-erbα*	20.0	-209	83.9	1.3313	0.3614	n.s.
	Npas2	26.0	-23	71.5	0.7656	0.2853	n.s.
	Cry1	20.0	-4	83.3	1.2854	0.3860	n.s
	Per2	22.4	-246	44.7	2.5486	1.2691	n.s.
	Clock	26.0	-7	15.1	1.4646	0.2051	n.s.
	Dbp	20.6	-240	60.0	1.2865	1.1543	n.s.
	Rev-erbβ	20.0	-209	83.9	1,3313	0.3614	n.s.
	Bmal1	25.3	-109	74.4	1.5788	0.6104	n.s.
	Rorα	20.0	-331	80.4	3.5973	1.4322	n.s.

Supplementary Table 6. Scoring criteria for the degree of colonic inflammation. NA, not applicable.

Scores / Criteria	0	1	2	3	4
Goblet cell loss	None	Mild	Moderate	Severe	NA
Mucosal thickening	None	Mild	Moderate	Severe	NA
Inflammatory cells infiltration	None	Mild	Moderate	Severe	NA
submucosa cell infiltration	None	Mild	Moderate	Severe	NA
Ulcers	0	0-25%	25-50%	50-75%	75-100%
Crypt abscesses	0	1-3	4-6	7-9	≥10

Supplementary Figure 1. Heatmap of relative mRNA expressions of inflammatory cytokines in mouse colon.

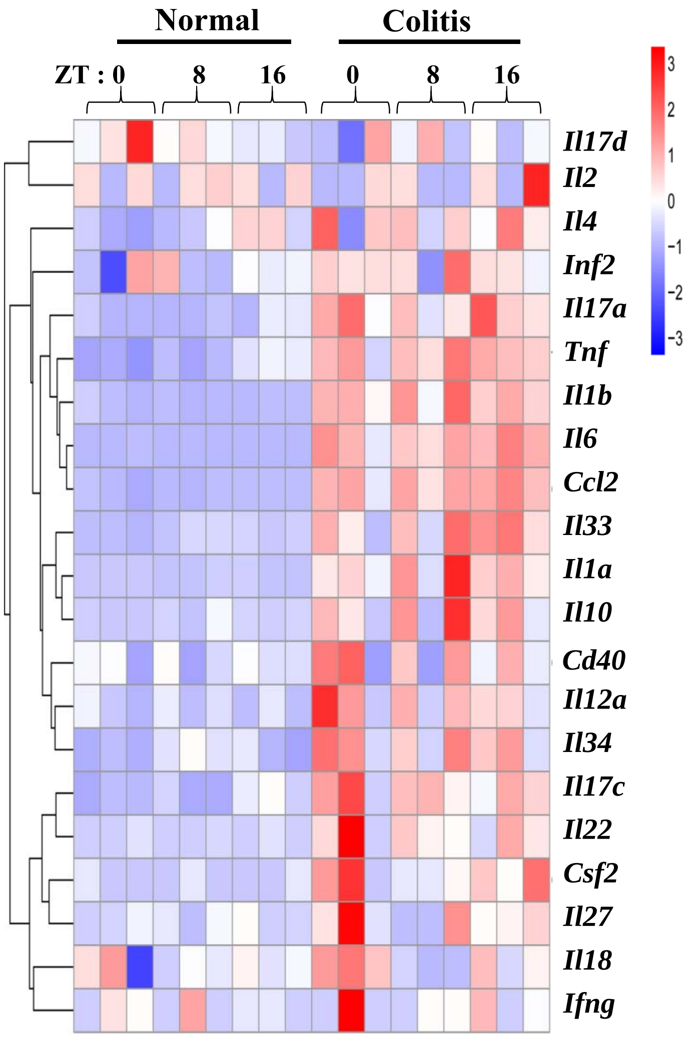

Supplementary Figure 2. Physiologic disruption of circadian clock exacerbates experimental colitis. (A) Representative actograms for the wheel running activities of mice subjected to regular LD12:12 (top panel) and jet lag (bottom panel) in three consecutive days. (B) Weight loss measurements of control and jet-lagged mice treated with DSS. Data are mean ± SD (*n* = 8). *p< 0.05 versus control at individual time points. (C) DAI scores of control and jet-lagged mice treated with DSS. Data are mean ± SD (*n* = 8). (D) Colon lengths of control and jet-lagged mice treated with DSS. Colon length was assessed at the time of necropsy. Data are mean ± SD (*n* = 8). (E) Representative micrographs for H&E staining of the colon. Scale bar = 100 μm. (F) Histopathological scores of control and jet-lagged mice treated with DSS. Data are mean ± SD (*n* = 8). (G) MPO activities of control and chronic jet-lagged mice treated with DSS on day 8. Data are mean ± SD (*n* = 8). For biochemical analyses, mice were sacrificed at ZT2 and the colons were collected. *P < 0.05 (t test or Mann–Whitney U test). JL, jet lag.

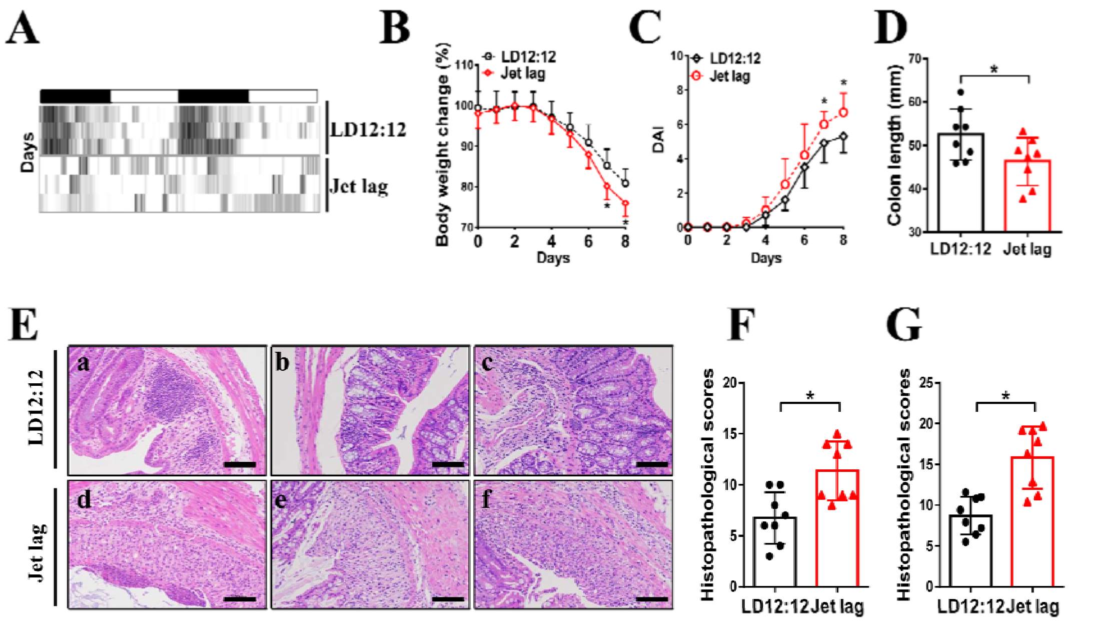

Supplementary Figure 3. Genetic disruption of circadian clock exacerbates experimental colitis. (A) Representative actograms for the wheel running activities of Bmal1 knockout mice subjected to regular LD12:12 (LD) or constant dark (DD). (B) PCR genotyping of the tails from wild-type mice (WT, +/+), heterozygotes (+/-), and homozygotes. The bands of 677-bp and 577-bp indicate WT and mutant alleles, respectively. (C) qPCR analyses of Bmal1 mRNAs in the colons from WT and Bmal1$^{-/-}$ mice. (D) Western blotting of Bmal1 protein in the colons from WT and Bmal1$^{-/-}$ mice at ZT2. (E) Weight loss measurements of WT and Bmal1$^{-/-}$ mice treated with DSS. Data are mean ± SD (n = 8). *p< 0.05 versus WT at individual time points. (F) DAI scores of wild-type and *Bmal1*$^{-/-}$ mice treated with DSS. Data are mean ± SD (n = 8). (G) Colon length measurements of wild-type and Bmal1$^{-/-}$ mice treated with DSS. Colon length was assessed at the time of necropsy. Data are mean ± SD (n = 8). (H) Representative micrographs for H&E staining of the colon. Scale bar = 100 μm. (I) Histopathological scores of wild-type and Bmal1$^{-/-}$ mice treated with DSS. Data are mean ± SD (n =8). (J). MPO activities of wild-type and Bmal1$^{-/-}$ mice treated with DSS. Data are mean ± SD (n =8). For biochemical analyses, mice were sacrificed at ZT2 and the colons were collected. *P < 0.05 (t test or Mann–Whitney U test).

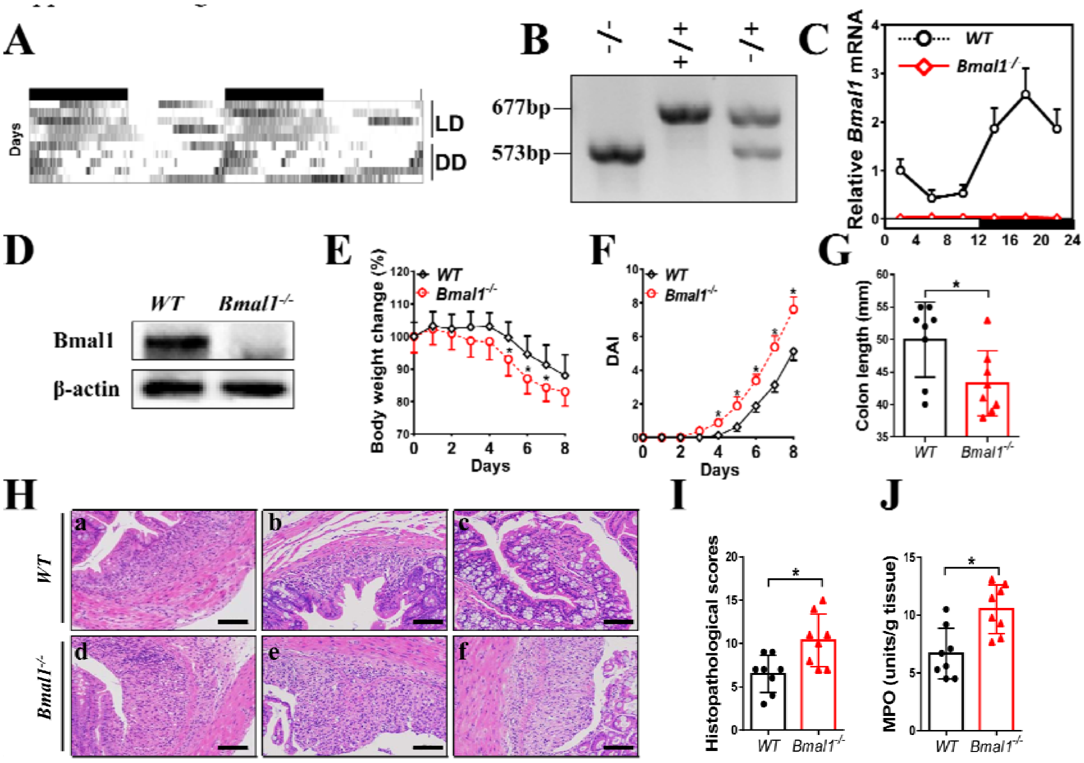

Supplementary Figure 4. Establishment and validation of Rev-erbα knockout mice using CRISPR/Cas9 technique. (A) Schematic diagram of the gene sequence for *Rev-erbα* showing the targeted region (between black arrows) for genetic deletion by the CRISPR/Cas9 technique. Green arrows indicate the positions of primers for PCR genotyping. (B) PCR genotyping of the tails from wild-type mice (WT, +/+), heterozygotes (+/-), and homozygotes (-/-). The bands of 516-bp and 920-bp indicate wild-type and mutant alleles respectively. (C) Western blotting of Rev-erbα protein in colons from WT and Rev-erbα$^{-/-}$ mice (ZT2).

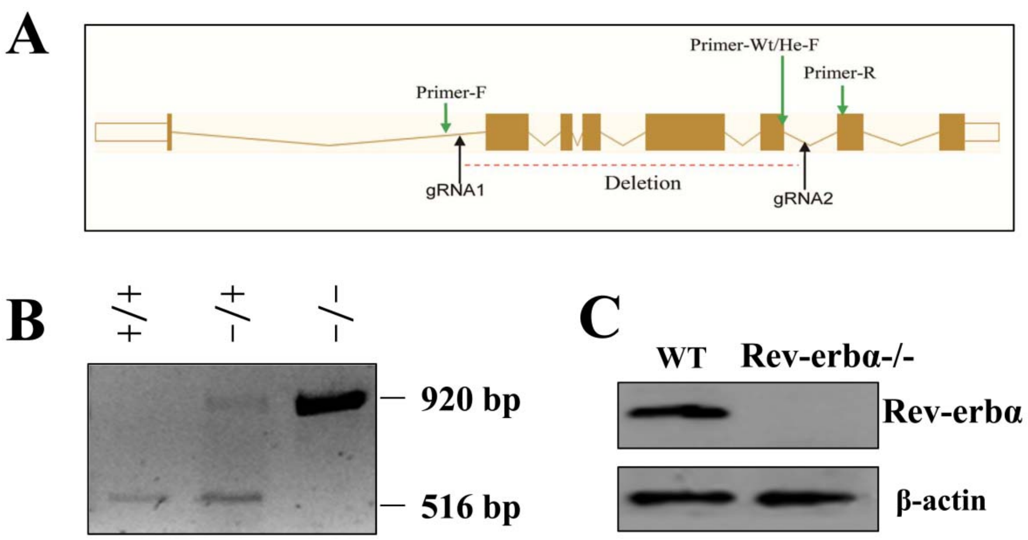

Supplementary Figure 5. qPCR assays on circadian and inflammatory-related genes in livers from WT mice. Data are mean ± SD (n = 5).

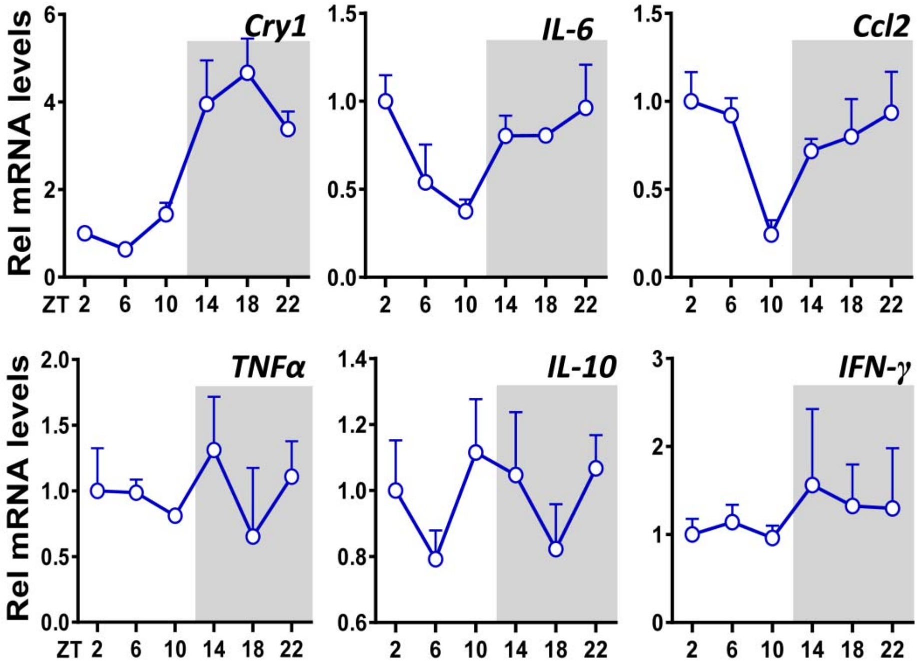

Supplementary Figure 6. (A) mRNA expressions of IL-18 in RAW264.7 cells treated with a Rev-erbα agonist (SR9009 or GSK4112), Rev-erbα siRNA or Rev-erbα plasmid. (B) ELISA measurements of IL-18 in supernatant of PMs. PMs were pretreated with SR9009 or vehicle for 1-h and then stimulated with LPS for 12-h and ATP for 30-min (added last). (C) Western blotting of Nlrp3, IL-1β and β-actin in BMDMs. BMDMs were stimulated with LPS for 12-h and ATP for the last 0.5h. The blot shown is representative of three independent experiments. (D) mRNA expressions of TNFα in RAW264.7 cells treated with vehicle or SR9009. A concentration of 10 μM was used for Rev-erbα agonists. Data are mean ± SD (n = 5). *P < 0.05 (Mann–Whitney U test).

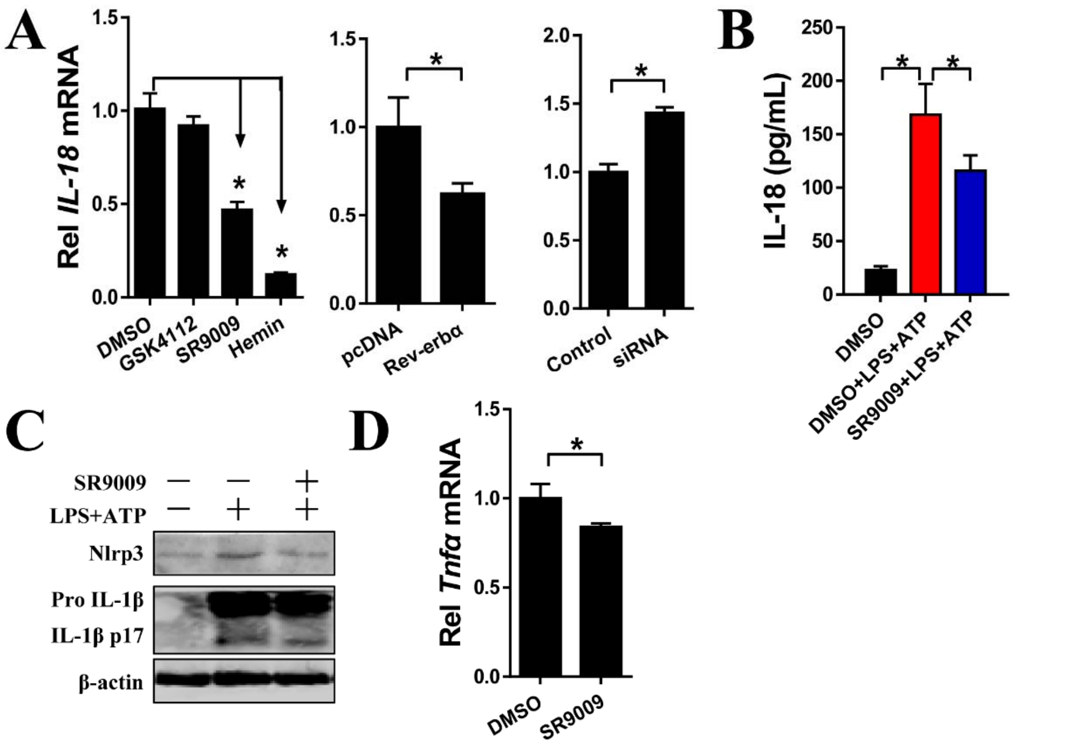

Supplementary Figure 7. (A) EMSA assay results, showing an interaction of Rev-erbα with Nlrp3-RevRE. The assays were performed with labeled Nlrp3-RevRE probes or labeled Bmal1-RevRE probe as indicated in the presence of nuclear extracts or probe competitors. (B) EMSA assay results, showing an interaction of Rev-erbα with p65-RevRE.

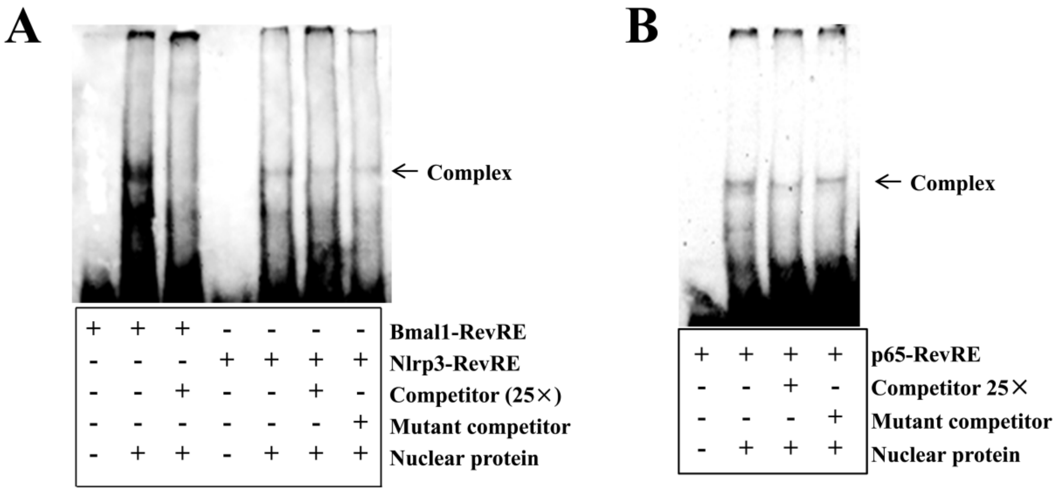

Supplementary Figure 8. (A) mRNA expressions of *p65* and *p50* in RAW264.7 cells measured by qPCR. The cells were treated with Rev-erbα siRNA (24-h) or Rev-erbα plasmid (24-h) or SR9009 (1-h), followed by stimulation with LPS (8-h). The concentrations of SR9009 and LPS for cell treatment were 10μM and 100 ng/ml, respectively. Data are mean ± SD (*n* = 5). *P < 0.05 (Mann–Whitney U test). (B) Schematic diagram of p65 luciferase plasmids with normal or mutated p65 promoter.

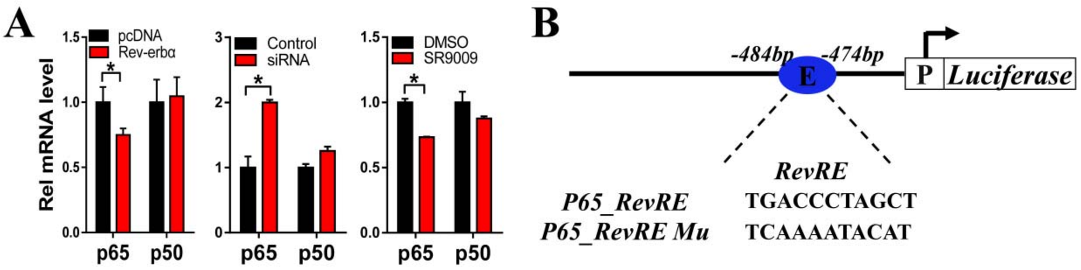

Supplementary Figure 9. SR9009 fails to alleviate DSS-induced colitis in Rev-erbα-deficient mice. (A) Weight loss measurements of SR9009- or vehicle-treated Rev-erbα$^{-/-}$ mice with DSS feeding. Data are mean ± SD (*n* = 8). (B) DAI scores of SR9009- or vehicle-treated Rev-erbα$^{-/-}$ mice with DSS feeding. Data are mean ± SD (*n* = 8). (C) Survival rates of SR9009- or vehicle-treated Rev-erbα$^{-/-}$ mice with DSS feeding. (D) Colon lengths of SR9009- or vehicle-treated Rev-erbα$^{-/-}$ mice with DSS feeding. Colon length was assessed at the time of necropsy. Data are mean ± SD (*n* = 6 for vehicle group, *n* = 5 for SR9009 group). (E) Representative micrographs for colon H&E staining. Scale bar = 100 μm. (F) Histopathological scores of SR9009- or vehicle-treated Rev-erbα$^{-/-}$ mice with DSS feeding. Data are mean ± SD (*n* = 6 for vehicle group, *n*=5 for SR9009 group). (G) MPO activities of mouse colons on day 8. Data are mean ± SD (*n* = 6 for vehicle group, *n* =5 for SR9009 group). (H) qPCR analyses of *Nlrp3*, *IL-1β* and *IL-18* expressions in whole colon tissues of mice with colitis on day 8. Data are mean ± SD (*n* = 6 for vehicle group, *n*=5 for SR9009 group). (I) ELISA measurements of colonic IL-18 on day 8 after DSS feeding. Data are mean ± SD (*n* = 6 for vehicle group, *n*=5 for SR9009 group). (J) Western blotting of Nlrp3, IL-1β and β-actin in colons from mice with colitis on day 8. The blot shown is representative of three independent experiments. SR9009 (50 mg/kg) was administered to mice via intraperitoneal injection once daily at ZT8 for 7 days prior to DSS treatment, and SR9009 dosing was continued along with DSS treatment. *P < 0.05 (t test or Mann–Whitney U test). SR, SR9009.

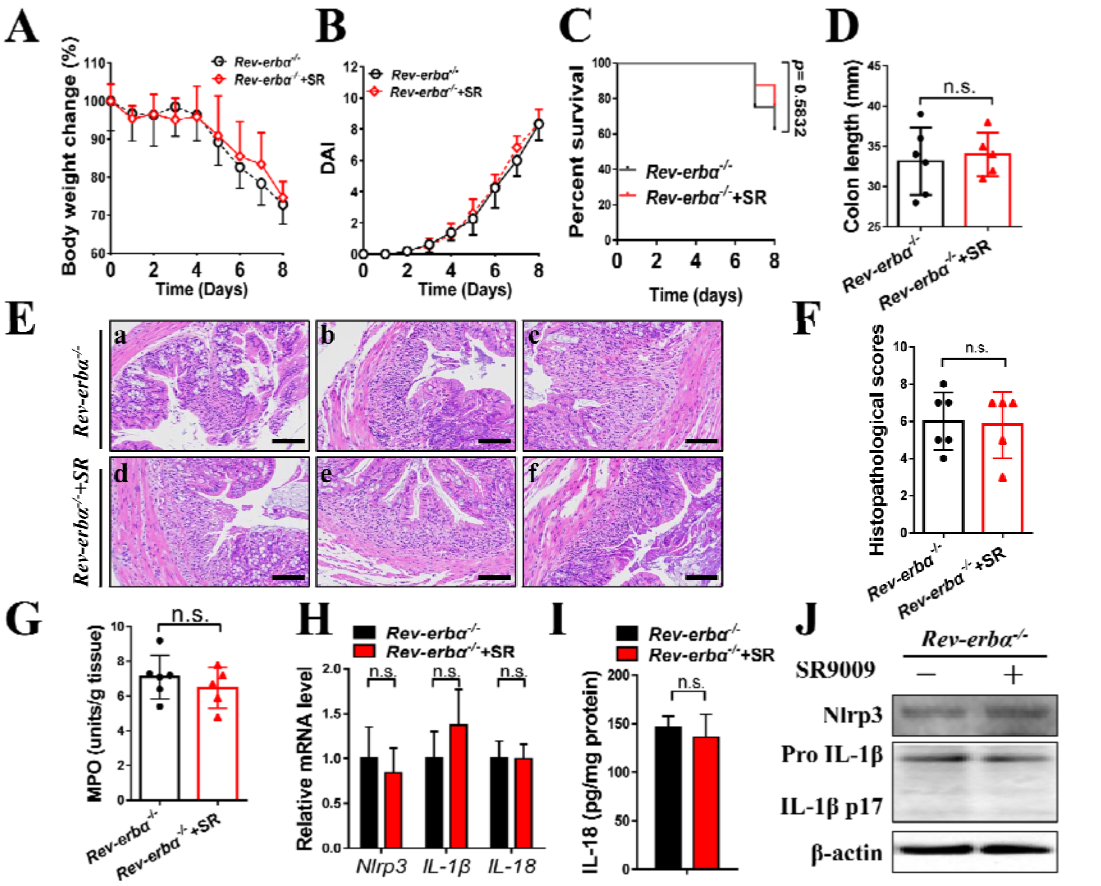

Supplementary Figure 10. (A) Circadian protein expression of Rev-erbα in mouse (WT) livers. (B) Animal experimental protocol for SR9009 administration prior to colitis induction. (C) Animal experimental protocol for SR9009 administration after colitis induction (n=8). Weight loss measurements of WT and Rev-erbα$^{-/-}$ mice treated with DSS. Data are mean ± SD (n = 8). *p< 0.05 versus vehicle- treated mice at individual time points (Mann–Whitney U test). In all experiments, SR9009 (50 mg/kg) was administered to mice via intraperitoneal injection once daily at ZT8.

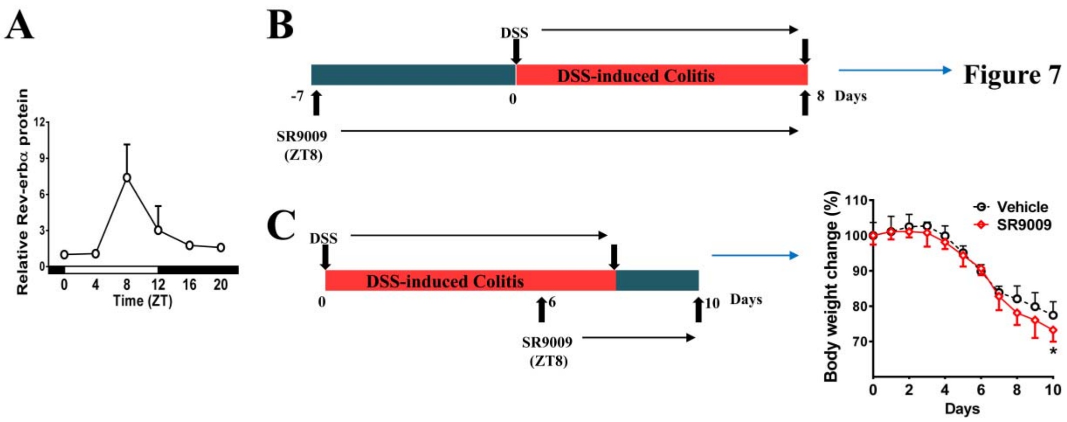

Supplementary Figure 11. qPCR measurements of *Nlrp3*, *IL-1β*, *p65* and *Nr1d2* in Raw264.7 cells. The cells were transfected with Nr1d2 (Rev-erbβ) plasmid 1for 24-h and then stimulated with LPS for 8-h. Data are mean ± SD (*n* = 3).

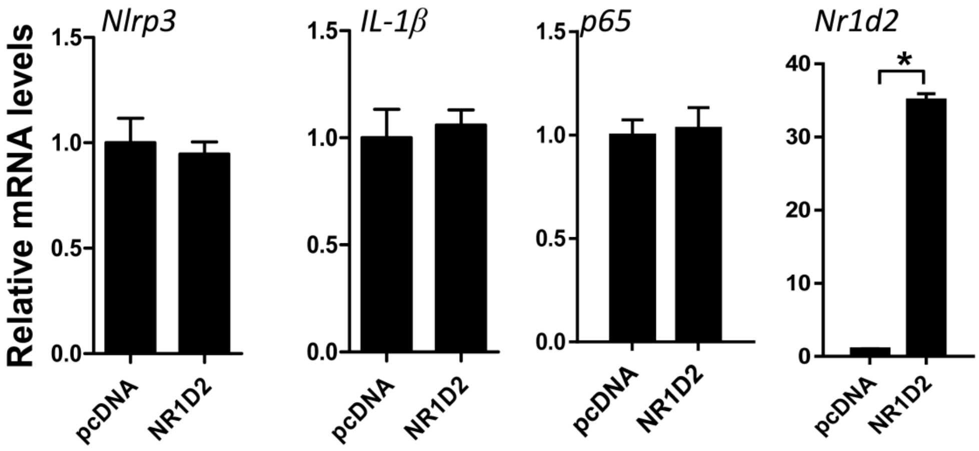

Supplementary Figure 12. Quantification data generated from Western blots in this study. Data are mean ± SD. *p< 0.05. n.a., not applicable.

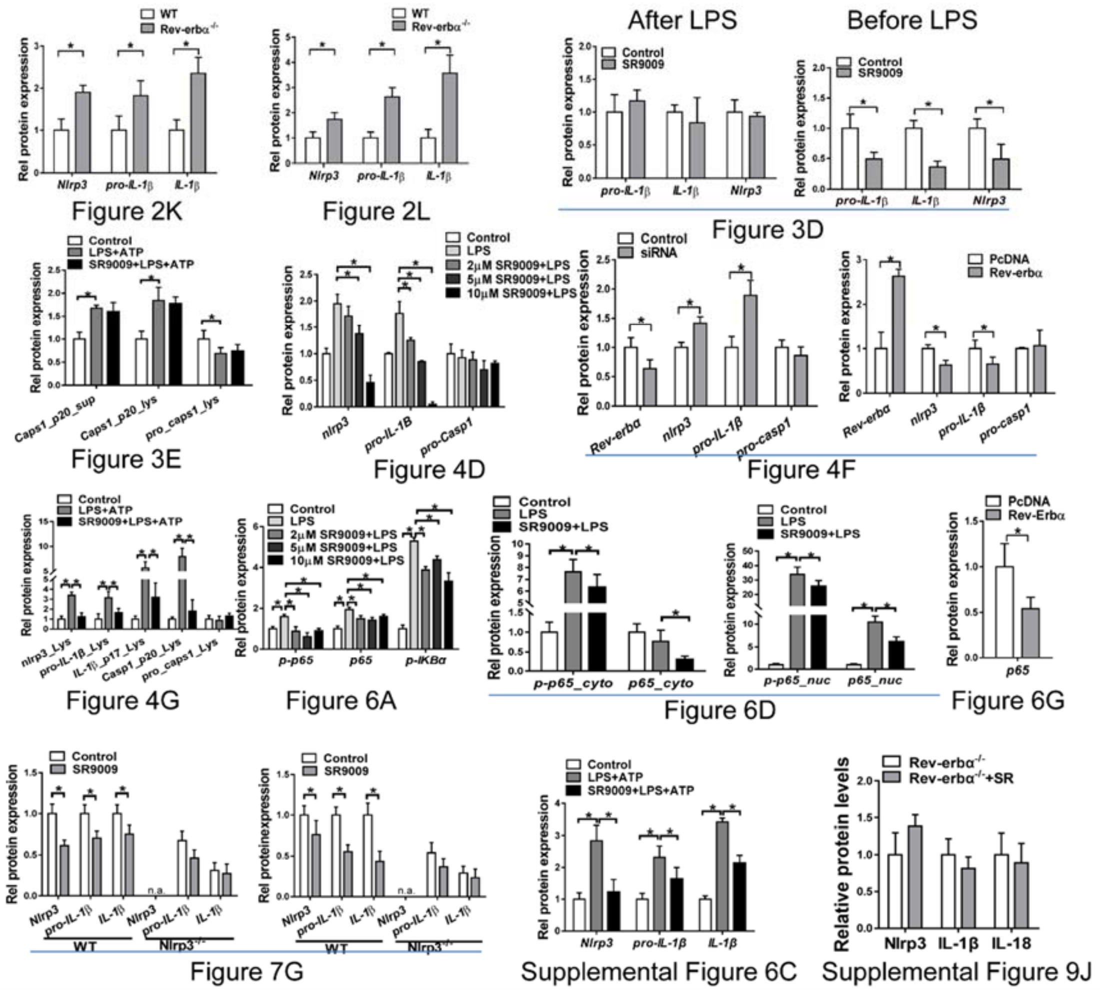

Supplementary Figure 13. Uncropped scans of representative Western blots.

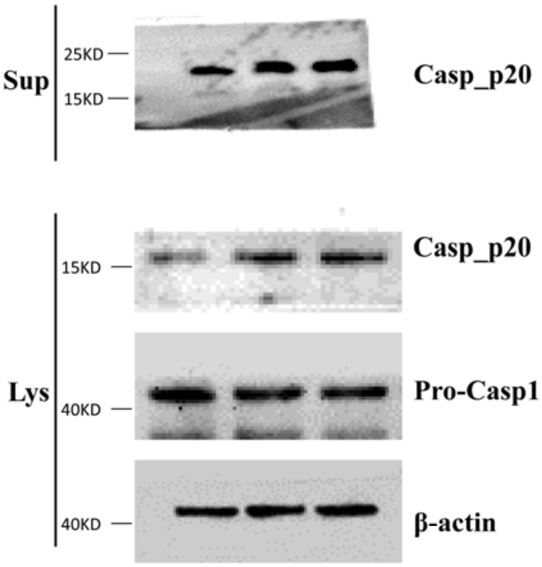

Figure 3E

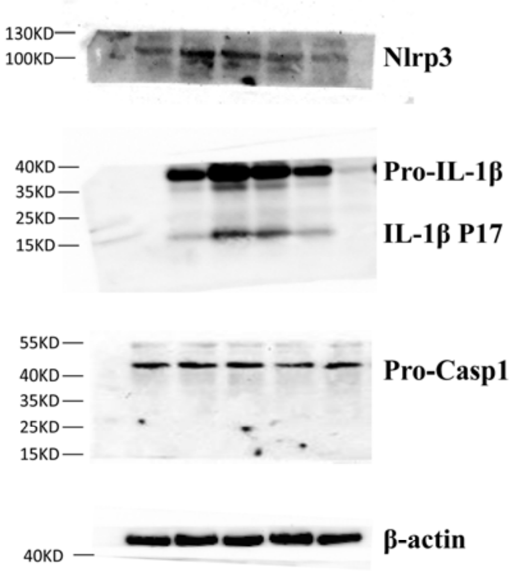

Figure 4D
